# Headaches of Sinus Origin

**Published:** 1919

**Authors:** 


**Headaches of Sinus Origin.** By WILLIAM IBBOTSON. *The Practitioner*, September, 1918.

One of the commonest causes of headache, but one frequently missed by medical men and ignorantly neglected by patients, is inflammation affecting one or more of the nasal or mastoid sinuses. There is a necessity for a more careful examination of the nasal, oral and aural regions in cases of headache of doubtful origin.

The ethmoidal, maxillary, frontal, sphenoidal and mastoid sinuses, taken in their respective groups, are closely related.

*Position and Character of Headache in the Above Regions*:

1. Acute ethmoidal sinusitis.

Character: dull, aching, often throbbing. Feeling of heaviness over bridge of nose. More or less constant.

Position: (*a*) Frontal, chiefly on the same side as the lesion, especially if anterior set of ethmoidal cells be involved. (*b*) Occipital if posterior set of ethmoidal cells be involved.

2. Chronic ethmoidal sinusitis.

Character: As in (1) but much more inconstant and less acute.

Position: As in (1).

3. Acute maxillary sinusitis.

Character: (*a*) Throbbing over site of inflammation. (*b*) Acute neuralgia following the course of superior maxillary nerve after it has entered the inferior or orbital fissure.

Position: On same side as lesion, pain referred to incisor and canine teeth, to premolar teeth, to lower eyelid, to side of nose, and to cheek and upper lip.

4. Chronic maxillary sinusitis. (*a*) Super-orbital, of same side, aching, and sometimes throbbing. (*b*) Frontal, generally on same side as lesion, but may be bilateral. (*c*) Over site of inflammation. (*d*) Occipital rarely.

5. Acute frontal sinusitis. Generally very intense. (*a*) Frontal, sense of fullness and tension, with decided throbbing. Pain more or less constant. Extreme tenderness over anterior wall of sinus. (*b*) May extend to the opposite side of frontal region. (*c*) Retro-ocular. (*d*) Vertical. (*e*) Occipital. (*f*) Anterior temporal.

6. Chronic frontal sinusitis. Much the same as in (5), but the pain is not so constant.

7. Acute sphenoidal sinusitis. (*a*) Occipital, aching. (*b*) Frontal. (*c*) Retro-orbital and probably due to retro-bulbar neuritis.

8. Chronic sphenoidal sinusitis. Very much as in (7), but much less constant.

9. Acute mastoid sinusitis. (*a*) Mastoid, neuralgic. (*b*) Temporal. (*c*) Along the jaw of the same side.

10. Chronic mastoid sinusitis. (*a*) Mastoid, aching and boring. (*b*) Frontal, of same side. (*c*) Parietal, of same side. (*d*) Occipital of same side.

Every case of persistent headache should have not only the eyes inspected, but also the nasal accessory and mastoid sinuses.

In many cases there are ocular lesions to account for the symptom of headache, but in a goodly proportion of them these ocular lesions are not the primary cause, but are secondary to disease of one or more of the above sinuses.

Influenza is a disease which, at the present time, is claiming many victims in Europe, and the author does not think that there is any disease more calculated to leave behind it such symptoms as headache, neuritis, etc.

The treatment for headaches of sinus origin is summed up in one word, — drainage.

